# How far have we come? From understanding collagen disorders to advancing treatment strategies

**DOI:** 10.1038/s41434-025-00572-3

**Published:** 2025-11-05

**Authors:** James Williamson, Yan Yu Chong, Wai Kit Hung, Joanna Jacków-Malinowska

**Affiliations:** 1 St John’s Institute of Dermatology, King’s College London, London, SE1 9RT UK; 2 Faculty of Medicine, The Chinese University of Hong Kong, Ma Liu Shui, Hong Kong SAR, China

**Keywords:** Gene therapy, Molecular biology

Collagen proteins play a vital role in numerous processes throughout the body, contributing to the function of various tissues. Consequently, pathogenic variants in genes encoding collagens are linked to a similarly diverse range of disorders, often with significant and severe effects.

Developments in gene editing now show promising avenues to tackle the root cause of these diseases in previously inconceivable ways. Kocsy et al., in their review [1], describe the diverse methods being investigated to target collagen disorders at a genetic level, their challenges, and new investigations that may resolve these issues. Exciting developments bringing the first gene editing therapies to patients may illuminate paths to take the discussed approaches to clinic [2–4].

**Fig. 1:**
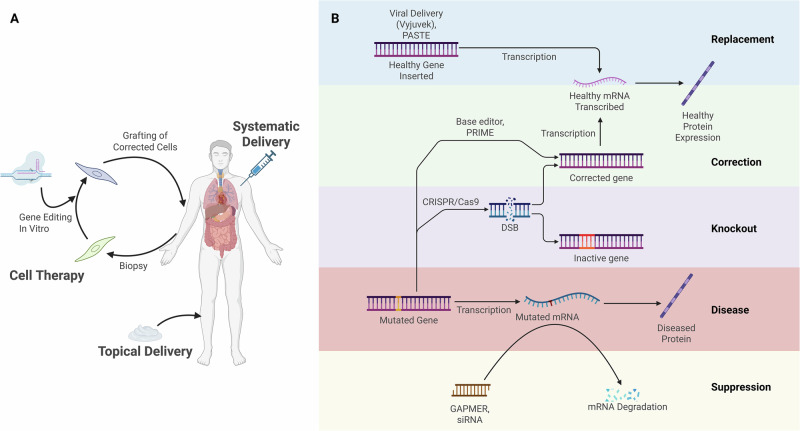
Gene editing approaches for personal medicine. **A** Showing delivery routes taken in gene editing treatments, indicating cell therapy, topical application, and systematic delivery. **B** A schematic of approaches taken in the treatment of genetic variants showing gene replacement, correction of pathogenic variants, knockout of pathogenic gene, disease without genetic intervention, and gene suppression at an RNA level, each annotated with relevant techniques. Created in BioRender.

The genetic causes of collagen disease can be tackled via several approaches (Fig. 1). Perhaps the most intuitive is to correct disease-causing variants at a genomic level, with many corrective methodologies for pathogenic collagen variants highlighted in this review. The emergence of CRISPR/Cas9 marked a turning point in genetic medicine, offering a versatile tool with the promise of editing disease-causing pathogenic variants. Traditional CRISPR/Cas9 uses RNA to guide Cas9 nuclease to cut a double stranded break (DSB) in target DNA, which can be combined with a donor template encoding the desired sequence. Homology directed repair (HDR), active in dividing cells, can then recombine the DSB, integrating the desired sequence. However, DSBs can risk random insertions/deletions (INDELS) of nucleotides at the cut site and induce apoptosis *via* p53 activation. By modifying Cas9 to nick a single strand of DNA, alternative gene editing tools have been created, further developed by fusing this nCas9 to other proteins. One such method is base editing, allowing biochemical conversion of individual nucleotides to correct variants [5, 6]. Prime editing provides a more versatile tool to correct disease variants, combining nCas9 and a reverse transcriptase, able to precisely integrate edits tens of bases long [7].

Alongside the development of new technologies, reapplying existing ones also show promise. In this way, Cas9’s DSBs have been used to induce INDELs, selectively knocking out pathogenic variants [8, 9]. The desire to silence pathogenic variants has also inspired investigation into non-CRISPR approaches, including siRNA [10] and GAPMER [11], antisense oligonucleotides which prevent transcription of pathogenic transcripts by complementarily binding mRNA.

Though many of the approaches covered by Kocsy et al. are effective in vitro, the expression patterns of each collagen come with unique requirements in therapeutic delivery. Gene editing treatments can be administered in vivo (applied topically or systematically), or ex vivo, with cells treated outside the body and then re-grafted into patients (Fig. 1A). The choice of gene delivery system depends on factors like cargo size (DNA, RNA, or protein), the desired duration of cargo expression (transient or genomic integration), and whether the target cells are dividing or non-dividing. Various viral vectors have seen application based on these factors [3, 12, 13], but concerns of immunogenicity and tumourgenicity of integrating viruses have led to recent clinical use of non-viral gene editor delivery, such as electroporation [4] and lipid nanoparticles (LNPs) [2].

While these approaches remain subjects of much research, gene replacement – introducing a healthy copy of diseased genes to recover protein expression – has successfully reached patients with positive results [3]. For clinical gene therapy, Vyjuvek’s approval for dystrophic epidermolysis bullosa (DEB) treatment marked a pioneering moment. It is the first topical gene therapy approved by the Food and Drug administration (FDA) (2023) and the European Medicines Agency (EMA) (2025), utilising a modified herpes simplex virus (HSV-1) to deliver functional *COL7A1* genes in vivo. Other therapies in clinical trial use lentiviral vectors: one approach uses edited keratinocytes to create epidermal grafts with functional *COL7A1* for transplantation (NCT01263379) [12], while another administered intradermal injections with lentiviral vectors containing the full-length *COL7A1* cDNA (NCT02493816) [13]. Both demonstrated robust safety profiles and promising improvements to wound healing, paving the way for more benchside-to-bedside transition to transform current treatments.

Despite these early successes, challenges linger and most novel technologies described in Kocsy et al. struggle to translate from preclinical promise to therapeutic reality. Gene replacement dominates the clinical pipeline, and while exciting in potential, precise editing therapy for collagen disorders have yet to leave the bench. In other fields, CRISPR programming is quickly proving to drive future advancements, with recent breakthroughs cementing its role in treating rare genetic diseases. The first approved CRISPR-based therapy, Casgevy, has established durable clinical benefits in sickle cell disease patients through targeted DNA modification [4]. Notably, a personalised base editor therapy was developed for an infant with neonatal-onset carbamoyl-phosphate synthetase 1 (CPS1) deficiency, successfully correcting a point variation and improving patient prognosis [2]. The process from diagnosis to delivery was realised within 8 months, highlighting how precise editing treatments can be developed with remarkable speed, provided the right resources are available. Why, then, is it so difficult to replicate these clinical achievements in collagen disorders?

Some critical hurdles include low efficiency, delivery system limitations and off-target effects. *COL6A1* silencing in Ullrich congenital muscular dystrophy (UCMD) showed low editing efficiencies (<1%), underscoring the challenges of HDR-dependent correction [7]. The large size of collagen genes (e.g. *COL7A1* spans 118 exons [14]) also represent a biological hurdle for delivery systems, as adeno-associated viruses (AAVs) have limited packaging capacity. Meanwhile, off-target effects like bystander edits further complicate therapeutic precision [15].

Yet, the field is also at a critical juncture. Adenine base editors (ABEs) represent a major advancement in precision gene editing by enabling targeted A-T to G-C conversions within a defined window specified by a single guide RNA. In particular, ABE8e can achieve 94.6% correction efficiency of the pathogenic *COL7A1* variant in DEB fibroblasts [5]. This opens a promising avenue to bypass current hurdles, creating potential for new innovative gene therapies.

Despite numerous technological advancements in gene therapy overcoming biological challenges, a significant gap exists in the number of clinical trials for collagen disorders. This suggests that the lack of trials stems from factors such as risk aversion and unequal funding, rather than inherent scientific limitations. Most collagen disorders are rare diseases, and therapies are unlikely to produce enough revenue to justify their costly development, making drugs for such conditions “orphan drugs” [16]. In fact, Vyjuvek was designated an orphan drug by the EMA [17], and the struggle to attract investment and subjects for recruitment are limitations that other gene therapies must overcome to reach patients. With most current trials focusing on DEB [18], the field can advocate for more inclusive clinical pipelines to deploy leading-edge tools in other collagen disorders, matching the wave of gene editing therapies approved for non-collagen diseases.

Recent years have also seen numerous novel technologies appearing on the preclinical stage, laying the groundwork for more treatments to reach clinic (Table 1). CRISPR’s challenge in delivering large gene inserts has spurred development of a groundbreaking alternative – Programmable Addition via Site-specific Targeting Elements (PASTE). By combining prime editing with serine integrases, PASTE allows for seamless insertion of large collagen genes (e.g. *COL4A3* and *COL4A4*) while eliminating the risk of off-target effects from DSB and p53-mediated cell death. By enabling site-specific integration without viral vectors or DSBs, PASTE provides a safer substitute to viral-based gene replacement therapy for collagen disorders [19].

**Table 1:** Comparison of CRISPR-Cas9 with novel gene editing technologies.

Feature	CRISPR/Cas9	Base Editing	PASTE	OTI	RNA Editing
**Mechanism**	DSB-dependent INDELs or edits based on DNA template	Chemical base conversion	Site specific large DNA insertions	Topoisomerase-mediated SSBs	RNA nucleotide conversion (A → I, C → U)
**Precision**	Moderate (off- targets)	High (reduced off-target effects)	High (reduced off-target effects)	High (reduced off-target effects)	High (no DNA changes)
**Presence of foreign protein**	Yes (Cas9 nuclease)	Yes (Cas9 nickase)	Yes (Cas9/Cas12k)	No	No (endogenous ADAR enzymes)
**Key Advantage**	Proven for clinical success	No DSBs, high precision	Large gene capacity, tested in liver models	Allele-specific cleavage	Reversible, no genomic risk
**Key Limitation**	Off-targets, PAM restriction	Limited base pair conversions	PAM restriction	Untested in collagen models	Transient effect, requires repeat dosing

Summarizing key features of current gene editing platforms including their molecular mechanisms, precision levels, requirement for foreign protein, primary advantages, and potential limitations.

Oligonucleotide-Topoisomerase Inhibitor (OTI) is another emerging tool to overcome a CRISPR limitation, namely its reliance on foreign proteins which may trigger immunogenicity. By coupling the specificity of an oligonucleotide to a topoisomerase inhibitor, this technology selectively cleaves DNA sequences without foreign proteins, thus minimising the risk of immunogenicity. This approach may be applicable for diseases such as osteogenesis imperfecta (OI), where OTIs show promise in correcting pathogenic point mutations in collagen genes like *COL1A1* while avoiding off-target edits in wild-type alleles [20].

Furthermore, RNA editing allows for transient correction by rewriting mRNA transcripts without irreversible genome alterations. By leveraging endogenous ADAR enzymes to catalyse adenosine to inosine conversions, it may be able to rescue faulty transcripts for disorders like UCMD without CRISPR’s genomic risks [21]. The continued development and clinical application of advanced gene-editing technologies hold immense promise for patients with collagen disorders. Through addressing current limitations and inefficiencies, these advancements pave the way for safer and more precise genetic therapies.

The field of gene therapy for collagen disorders has reached a pivotal point, with multiple laboratory breakthroughs now poised to transform patient care in the near future. Advanced novel tools such as base editors, PASTE, OTIs, and RNA editing have emerged as inventive alternatives to address the limitations of CRISPR to offer safer and more precise gene editing. As these technologies further advance into pre-clinical and clinical trials, such novel approaches have the potential to expand the reach of treatment for those suffering from genetic collagen disorders, effectively acting as a lifeline for patients. Alongside these developments, the advances to CRISPR and base editing is evidenced by their recent clinical successes, showing them to be safe and capable tools. If given the necessary resources, existing and novel technologies have the potential to bring forth an era of transformative genetic therapies for patients suffering from collagen disorders and other genetic diseases.
